# Associations between the objective and perceived food environment and eating behavior in relation to socioeconomic status among adults in peri-urban settings: results from the CIVISANO study in Flanders, Belgium

**DOI:** 10.1186/s12942-024-00369-4

**Published:** 2024-05-09

**Authors:** Yasemin Inaç, Suzannah D’Hooghe, Delfien Van Dyck, Sarah Dury, Stefanie Vandevijvere, Benedicte Deforche, Eva M. De Clercq, Nico Van de Weghe, Karin De Ridder

**Affiliations:** 1 Sciensano, Department of Epidemiology and Public Health, Brussels, Belgium; 2 Faculty of Medicine and Health Sciences, Department of Public Health and Primary Care, Ghent University, Ghent, Belgium; 3 Faculty of Psychology and Educational Sciences, Adult Educational Sciences, Vrije Universiteit Brussel, Brussels, Belgium; 4 Faculty of Sciences, Department of Geography, Ghent University, Ghent, Belgium; 5 Faculty of Physical Education and Physiotherapy, Department of Movement and Sport Sciences, Vrije Universiteit Brussel, Brussels, Belgium; 6 Faculty of Medicine and Health Sciences, Department of Movement and Sports Sciences, Ghent University, Ghent, Belgium; 7 Sciensano, Department of Chemical and Physical Health Risks, Brussels, Belgium; 8 Society and Ageing Research Lab (SARLab), Vrije Universiteit Brussel, Brussels, Belgium

**Keywords:** Food environment, Eating behavior, Food outlets, Socioeconomic status, Peri-urban settings

## Abstract

Obesity, a significant public health concern, disproportionately affects people with lower socioeconomic status (SES). Food environments have been identified as part of the causal chain of this disparity. This study investigated variations in the food environment across groups with different SES profiles residing in peri-urban municipal settings. In addition, it examined the association of the perceived and objective food environments with eating behaviour and assessed if these associations were moderated by SES. Utilizing GIS and survey data (*n* = 497, aged 25–65), results showed differences in the objective and perceived food environments based on SES. Respondents with higher SES perceived their food environments as better but resided farther from all food outlets compared to respondents with lower SES. However, there was no difference in outlet density or mRFEI between SES groups. SES moderated associations between the objective and perceived food environments and most eating behavior outcomes except fast food consumption frequency. For fruits and vegetables, SES moderated the association between neighborhood availability and consumption frequency (β0.23,CI0.03;0.49). Stratified analysis revealed a positive association for both lower (β0.15, CI0.03;0.27) and higher (β0.37, CI 0.12;0.63) SES groups. For snack foods, SES moderated the association between healthy outlet density and consumption frequency (β-0.60, CI-0.94; -0.23), showing statistical significance only for respondents with higher SES (β0.36,CI 0.18;0.55). Similarly, for sugar-sweetened beverages, a statistically significant interaction was observed between unhealthy outlet density in the 1000m buffer and consumption frequency (β 0.06, CI 0.02; 0.11). However, this association was only statistically significant for respondents with higher SES (β-0.02,CI -0.05;-0.0002). These results emphasize the significance of SES as a crucial element in comprehending the connection between the food environment and eating behaviour. Indicating the need for policymakers to take SES into account when implementing food environment interventions, particularly when focusing on the neighborhood food environment without considering residents’ SES and their perceptions.

**Supplementary Information:**

The online version contains supplementary material available at 10.1186/s12942-024-00369-4.

## Background

Noncommunicable diseases (NCDs) are the leading cause of death and a major disease burden worldwide [1]. In 2022, NCDs accounted for approximately 74% of mortality worldwide [2]. Overweight and obesity are considered one of the main causes of NCDs [3]. Currently, the rates of overweight and obesity are high and are still rising [4], and poor eating behavior is considered one of the main causes [5]. The food environment is considered a primary driver of eating behavior [6].

According to the Model of Community Nutrition Environments developed by Glanz et al. (2005), the food environment is a multidimensional concept that includes four types of nutrition environments: [1] the community nutrition environment (e.g. the type, location and accessibility of food outlets) [2], the consumer food environment (e.g. the availability of healthy options, the price, promotion and shelf placement of foods) [3], the organizational food environment (e.g. access to (healthy) foods in workplaces and educational settings) [4], the information environment (e.g. food advertisements in the media) [7]. In recent years, the digital food environment - which has been defined by the WHO as online settings through which flows of services and information that influence the food and nutrition choices and behaviours of people is directed - has also attracted considerable attention due to food-delivery apps [8]. The food environment can be captured using objective measures, respondents’ perceptions, or a combination of both [9, 10].Objective measures can assess the accessibility and the availability of food outlets using Geographic Information Systems (GIS). Perceived measures capture respondents’ impressions of factors such as perceived availability and affordability of food options, as well as shopping experiences in their neighborhood [11, 12]. Studies that include both objective and perceived measures of food environment are scarce [13–15]. However, a cross-sectional study found that, although both types of measures were associated with eating behavior, the outcomes were not identical and might provide complementary information [16]. Thus, combining objective and perceived measures of the food environment offers the potential to form a more complete characterization of food availability and accessibility [17].

The food environment is context specific. Evidence from English-speaking countries suggests that people in rural communities face greater barriers to accessing fresh fruits and vegetables than do those in urban communities [18, 19]. In addition, data from the United States show that people with low SES in rural areas pay more for low-quality food than urban residents with high SES [20, 21]. Furthermore, the Belgian food environment may present different challenges compared to countries in the Anglosphere and other mainland European countries [22]. Compared to other European countries, the Flemish region (the northern Dutch speaking part of Belgium) is densely populated and characterized by high urban sprawl, that is, the expansion of urban areas into the surrounding agricultural areas with patchy and scattered urban development [23]. 39% of the Flemish population lives in peri-urban areas [24]. UNESCO defines these areas as zones of transition from urban to rural land, located between the outer limits of urban and regional centers and the rural environment [25]. These settings therefore by definition combine urban and rural characteristics [26].

Monofunctional, low-density neighborhoods with high travel distances to food outlets are prevalent in Flanders [27]. Previously, this was not seen as a problem because these neighborhoods were typically populated by highly mobile middle classes [28]. However, the phenomenon of peri-urban areas is increasing worldwide as urban sprawl spreads further into rural areas [24], resulting in a more heterogeneous socioeconomic composition among residents in peri-urban areas [29]. Residents with a lower socioeconomic status might be affected differently by the food environment in these areas. Previous research has reported that transportation barriers pose a substantial barrier for people with lower socioeconomic statuses to access healthy food [30, 31], whereby, households without car ownership are more likely to be food insecure, compared to those who do own a car [32]. The lack of car ownership compromises the ability to access food because people may have to rely on public transport such as buses whose schedules may be inconvenient, walk-along roads not suited for pedestrians or shop at food outlets that are easier to access but offer fewer choices at higher prices [31, 33]. Residents with a lower SES residing in Flemish peri-urban areas may experience higher travel times to food outlets by foot and/or bicycle, and car ownership may improve their ability to travel outside of their neighborhood to obtain food [34].

Taking the specific nature of peri-urban areas into account could improve our understanding of context-specific influences of the environment on eating behavior. To the best of our knowledge, only a limited number of studies in Europe (such as the Mont’Panier study by Recchia et al. (2022)) have examined the food environments of populations with distinct socioeconomic profiles in peri-urban settings and investigated variations between these groups [35]. In Belgium, the majority of research on nutritional inequities has focused on urban settings such as Brussels, Ghent, and Antwerp. However, nutritional inequities are not solely an urban phenomenon, and people with lower SES reside outside cities. Nevertheless, in peri-urban and rural areas compared to more urban ones, the degree of deprivation and notably it’s concentration are less pronounced as outlined in the study of De Decker & Goossens (1999) [36]. This reduced concentration of deprivation implies that it is less visible in these areas. Consequently, the decreased visibility of people living in deprivation in peri-urban settings may lead to a limited understanding of their food environment. Their diminished visibility potentially translated into less studies that specifically examine the food environment of this group in peri-urban areas. Therefore, policies aimed at improving the food environment in these settings might be unsuccessful, because they are not aligned with the real-life environments of people with lower SES in peri-urban areas.

Building on the above-mentioned literature, this study will (1) assess whether the food environment varies between groups with distinct socioeconomic statuses in peri-urban settings (2), examine the associations between both the objective and perceived food environment domains and eating behavior, and (3) investigate whether socioeconomic status moderates the (potential) associations between the food environment and eating behavior.

## Setting

Since the definition of peri-urban areas from UNESCO is rather vague, peri-urbanity was operationalized based on several criteria within this study. First, population size was taken into account (< 50.000 inhabitants) because a population size of > 50.000 is consistent with a city, and because of the urban character of cities, these were immediately excluded [37]. Ninety-three municipalities were selected based on the population size. In addition to population size, socioeconomic indicators for municipalities (e.g., exclusion of industrial areas, richest municipalities, and municipalities with a high number of elderly residents) from the Belfius Index were also considered [38]. This was done to ensure that a representative mix of respondents from different socioeconomic groups and within the age range of 25 to 65 years could be sampled from the selected municipalities. This reduced the number of eligible municipalities to 37. After considering the feasibility of the research team to commute to these municipalities (less than two hours travel one way), 12 municipalities remained eligible. After contacting these 12 municipalities, two (Duffel and Herselt) decided to participate in the project. These municipalities were further assessed in ArcGIS using the European Degree of Urbanization classification (DEGURBA). Although the DEGURBA classification does not have a peri-urban definition, it classifies areas as (1) cities, (2) towns, (3) suburbs, (4) villages, (5) dispersed rural areas, and (6) mostly uninhabited areas [39]. To align with the definition of UNESCO, it was decided to focus on municipalities classified as towns, since the DEGURBA manual described towns and semi-dense areas as being between cities and rural areas. The participating municipalities (Duffel and Herselt) were classified as towns, according to the DEGURBA classification.

Within these selected peri-urban municipalities, some respondents lived in more central parts of the municipalities (the village center or core), while others resided further away from the village center (e.g., towards the edges of the municipalities).

## Materials and methods

In this cross-sectional study, data from respondents in the CIVISANO project was used, a mixed-methods study investigating the role of objective and perceived environmental factors on physical activity and eating behavior among adults residing in two medium-sized peri-urban municipalities, Duffel and Herselt, in Flanders, Belgium [40]. The study was approved by the Medical Ethics Committee of Ghent University Hospital (BC-248 09260) and conducted in accordance with the recommendations of the Belgian Data Protection Authority. All the respondents provided informed consent.

The study population consisted of respondents to the questionnaire part of the project. The questionnaire included items on sociodemographics, eating behavior, health behavior, and perceptions of the local environment. It was primarily based on the Local Health Interview Survey (Local HIS) of 2019 [41]. Variables included in the Local HIS 2019 were derived from the Belgian National Health Interview Survey of 2018 [42]. Additionally, items from the Flemish version of the Sustainable Prevention of Obesity through Integrated Strategies Project (SPOTLIGHT) and the Perceived Nutrition Environments Measures Survey (NEMS-P) were included to assess the food environment [43, 44]. To increase accessibility, the questionnaire was read and redacted by Wablieft, a Flemish organization that advocates the accessibility and comprehensibility of the Dutch language for underserved groups [45]. Full details on the questionnaire and CIVISANO project can be found elsewhere [40].

### Study sample

The inclusion criteria for respondents were age between 25 and 65 years and residing in Duffel or Herselt. In the study, an overrepresentation of respondents with lower socioeconomic status was intended. Therefore, active recruitment, similar to time-location-based sampling, was used. The locations (e.g., food banks/distributions, neighborhoods with a higher concentration of government-assisted social housing, private rentals, and remedial schools.) in which people with lower socioeconomic status were overrepresented were compiled into a list, and these locations were randomly visited by volunteers during the recruitment period (i.e., between May and November 2021). The volunteers offered respondents the option to fill in the questionnaire themselves using a tablet, or guided the respondents through the questionnaire using an interview approach. In conjunction, other sampling strategies were also employed, such as posting QR codes to fill in the questionnaire on traditional (local) media and social media and making the questionnaire available in local places that were not visited during the active recruitment, such as libraries and medical offices.

### Dependent variable – eating behavior

Respondents completed several short statements regarding their regular consumption frequency of key indicators of healthy and unhealthy food groups, such as fruits (excluding juices), vegetables (excluding juices and potatoes), sugar-sweetened beverages (excluding light and zero beverages), sweet and salty snacks, and fast food. These statements were based on the nutritional part of the Belgian National Health Interview Survey [42]. Statements included “How often do you eat or drink the following?” with examples of food and/or drinks provided to each group. The response options were: “never”, “less than once a week”, “1 to 3 times a week”, “4 to 6 times a week, “once a day”, and “more than once a day”. Subsequently, the responses were converted into numerical values corresponding to the times per day, as defined by Van Mierlo et al. (2021) and Haubrock et al. (2011) [46, 47]. In addition, fruit and vegetable consumption frequencies were combined into one variable. Resulting in a total of four outcome variables: fruit-and vegetable consumption frequency (FV), fast-food consumption frequency (FF), snack consumption frequency (SN) and sugar-sweetened beverages consumption frequency (SSB).

### Explanatory variables – food environment

The assessment of the food environment was conducted at the individual level for each respondent and was divided into objective and perceived domains. For the objective domain, multiple objective measures, that is, the proximity to healthy, unhealthy, and fast-food outlets, the density of healthy, unhealthy, and fast-food outlets in the 500m buffer, the density of healthy, unhealthy, and fast-food outlets in the 1000m buffer and the mRFEI in the 500m and 1000m buffer were assessed. To calculate these, respondents were asked to localize the intersection nearest to their home address when filling in the questionnaire. The nearest intersection, instead of the home address, was used to protect respondents’ privacy. Using ArcGIS Pro, the location of each respondent was linked to information regarding all food outlets in the municipalities. Data on these outlets were obtained from the Locatus 2020 database, supplemented with data on local (farmers) markets, farm stores, and community gardens. Solely, food outlets selling food as a primary function were included and outlets selling food as a secondary function, e.g. cinema’s or sport facilities with vending machines, were excluded. Food outlets were classified as healthy, neutral or unhealthy based on the opinion of an expert committee consisting of food policy experts and nutritionists from Flanders. From the unhealthy category, fast food outlets were extracted as separate metrics. Healthy food outlets were defined as outlets that primarily sell healthy foods, i.e. greengrocers, fishmongers, farmers’ markets, stores selling nuts and organic stores. Neutral outlets were defined as outlets selling a mix of healthy and unhealthy food and drinks, i.e. supermarkets, mini-supermarkets, poulterers, cheese shops, bakeries, hotels with restaurants, lunchrooms and full-service restaurants. Unhealthy food outlets were defined as outlets that primarily sold unhealthy foods, i.e. cafés, pancake restaurants, butchers, flan restaurants, ice cream parlors, confectionary and convenience stores. Fast food outlets were defined as outlets selling fast food to take-away and/or eat in, i.e. fast food outlets (sit-in and delivery) and grillroom/shawarma outlets.More information about the classification of food outlets can be found in Additional File 2 and in the study by Smets et al. (2022) [22]. In line with the classification, suggested by the expert committee, it was decided to classify supermarkets as neutral in this study, even though multiple studies have classified them as healthy (e.g. Thornton et al., 2012;Clary et al., 2016) [48, 49]. This was based on our research questions, which were formulated to evaluate the consumption frequency of multiple nutritional categories, not solely fruits and vegetables but also fast-food, snacks, and sugar-sweetened beverages, in relation to healthy and unhealthy food outlets. Unlike many studies that primarily focus on fruit and vegetable intake in relation to accessibility (e.g. Evans et al., 2012; Pessoa et al., 2015) [50, 51]. Additionally, although supermarkets are crucial points of access for fruits and vegetables, studies have shown that supermarkets may not universally be deemed as healthy food outlets. For instance findings from the International Network for Food and Obesity/NCDs Research, Monitoring, and Action Support (INFORMAS) (Vandevijvere et al., 2018) and a Belgian study from Vandevijvere et al. (2023) suggest that the ratio between healthy and unhealthy foods and drinks in supermarkets, is geared towards unhealthy foods [52, 53]. This is in line with in-store measurements, which have been conducted between February and May 2021, as part of this project. Which assessed the in-store food environment in a sample of six supermarkets across four different chains in the municipalities participating in this study. Overall, in the visited supermarkets the ratio between healthy/unhealthy foods was found to be 0.45, indicating that for every 10m of shelf length of unhealthy foods there was 4.5m of healthy foods. Based on the above-mentioned findings it was therefore decided to retain the classification of supermarkets as neutral.

Buffers of 500m and 1000m were used to calculate the density. These buffer sizes were chosen based on previous studies conducted internationally and in Ghent (Belgium) as part of the ‘International Physical Activity and Environment Network’ (IPEN) which recommend the use of street network buffers of 500m and 1000m around respondents’ residences to develop a standardized spatial definition of a ‘neighborhood’ that can be used to compare results between countries [54, 55]. In addition, two different buffer sizes were used to account for potential variations in the food environment and travel behaviors of respondents to food outlets [56].

Proximity measures were defined as the shortest road network distance in meters to the nearest healthy, unhealthy, and fast-food outlets and were calculated for each respondent. In addition, the modified Retail Food Environment Index (mRFEI), which is the ratio of healthy food outlets to the total number of food outlets, was also calculated as part of the objective measures. The mRFEI score is a continuous variable ranging from 0 to 1. A lower score indicated a more unhealthy food environment [57]. A score of 0 indicated that there were no healthy food outlets in an area. Because the mRFEI could only be calculated for respondents with food outlets in their buffers, respondents without healthy, neutral and unhealthy food outlets in their buffers were excluded (*n* = 71).

The mRFEI was calculated using the following formula:

$$mRFEI=\frac{\#\, healthy\,food\,outlets}{\begin{aligned}&\# healthy\,food\,outlets + \# neutral\,food\,outlets \\&+ \# unhealthy\,food\,outlets\end{aligned}}$$

The perceived domain was based on respondents’ perceptions of five statements regarding their local food environment, which were based on the NEMS-P questionnaire [44]. The statements included: “Fresh fruits-and vegetables are easily available in my neighborhood”, “Fresh fruits- and vegetables are cheap to buy in my neighborhood”, “Fresh fruits-and vegetables in my neighborhood are of good quality”, “Fast-food is easily available in my neighborhood” and “Fast-food is cheap to buy in my neighborhood”.

### Covariates

Respondents’ age and gender identity were included as covariates in all the models. Age was measured in years and rounded off to the nearest whole number. Respondents’ gender identity was determined by asking respondents to indicate if they identified as “male,” “female” or “prefer not to answer.” SES was used as an interaction term to assess its moderating effect on the associations between objective and perceived food environments and eating behavior. All respondents were classified as having a lower socioeconomic status (LSES) or higher socioeconomic status (HSES). LSES was based on meeting at least one of the following criteria: (a) low level of education (no tertiary level), (b) no current paid employment within the household, and (c) net family income below the national minimum income (that is, € 1625.72 gross per month per person in 2021), taking family size into account, (d) perceived financial difficulties (= difficult to very difficult to make ends meet on a monthly basis), or (e) low perceived socioeconomic status (lower than or equal to five on the MacArthur scale). The MacArthur scale measures subjective socioeconomic status by asking respondents to place themselves on a hypothetical ladder relative to others in their group [58]. All variables used as covariates were obtained from the questionnaire and were self-reported.

### Statistical analysis

#### Study population

Descriptive statistics were used to describe the characteristics of the total sample and were stratified by SES. Continuous variables are presented as means and standard deviations (SD). Categorical variables are presented as frequencies.

#### Differences in food environment along SES

Independent t-tests, chi-square, or Mann-Whitney U analysis tests were conducted to evaluate the hypothesized differences in domains in the food environment between SES groups.

#### Food environment, eating behavior and SES

Linear regression models were used to test associations between the objective and perceived food environment domains and the four eating behavior outcomes (i.e., FV, FF, SN, and SSB consumption frequency), adjusted for of age and sex. Before running the models the assumptions for linear regression were first tested. During this assessment, the assumptions were checked for using the original- and log-transformed values of the dependent variables to determine whether this resulted in more robust conclusions. Since there were no large differences when comparing the plots showing residuals vs. fitted values and both showed homoscedasticity, the original values of the dependent variables were used because it is known that log-transformed variables are more difficult to interpret [59]. Afterwards, we tested whether SES had a moderating effect on the association between domains of the food environment and eating behavior. Six multivariable models with interaction terms, that is, one model per domain (i.e., objective and perceived) for each eating behavior outcome under study were created. As indicators of the perceived domain were not available for SN and SSB consumption frequencies, only objective indicators were tested for these outcomes. Table S1 in Additional file 1 shows the variables tested for each eating behavior outcome. Stratified analysis of the SES groups was conducted when statistically significant interactions between domains of the food environment and eating behavior were observed. If no statistically significant interaction term was observed, the effect on the whole population was assessed and the analysis repeated without including an interaction term. These analyses were adjusted for the five variables used to construct SES (i.e., education, employment, income, subjective SES, and subjective financial difficulties). This was done to ensure that no confounding factors occurred when analyzing the main effects. The outcomes of the analysis of the main effects are shown in Table 1 in Additional File 5. Statistical tests were two-sided, and differences or associations were considered statistically significant at *p* < 0.05. All analysis were performed using RStudio. 

## Results

### Descriptive characteristics

Table 1 shows the descriptive characteristics of the respondents’ demographics, socioeconomic characteristics, and eating behaviors. In total, 498 respondents completed the questionnaire. The LSES and HSES groups consisted of 268 and 229 respondents, respectively. In the LSES group, 89 respondents met one of the LSES criteria; 75 respondents met two criteria; 32 respondents met three criteria; 39 respondents met four criteria and 33 respondents met five criteria. The mean age (SD) of the respondents was 45.5 (10.9) years. Of these, 66.6% were female and slightly more than half (64.2%) had completed higher education. The mean age (SD) of LSES respondents was 47.8 (11.1) years, while the mean age (SD) of the HSES respondents was 42.8 (10.0) years. Overall, LSES respondents consumed less fruit and vegetables but more fast food and sugar-sweetened beverages than HSES respondents. However, HSES respondents had a greater consumption frequency of snack foods than LSES respondents. The consumption frequency of both fruit and vegetables and sugar-sweetened beverages showed a statistically significant difference between the LSES and HSES groups.

**Table 1:** Demographic and socioeconomic characteristics of the total sample and by SES. Respondents in the CIVISANO-survey, Flanders, 2021

	Total sample (*n* = 498) Mean or %	LSES (*n* = 268)	HSES (*n* = 229)	Test statistic (i.e. T-test/Chi-square/Mann-Whitney U)	
**Demographics**					
Age	45.5 ± 10.9	47.8 ± 11.1	42.8 ± 10.0	< 0.001***^1^	
Gender identity				< 0.001***^2^	
Male	33.0	34.7	31.0		
Female	66.6	64.6	69.0		
Other	0.4	0.7	0		
**Socioeconomics**					
Educational level				< 0.001***^2^	
No education	0.4	0.7	0		
Primary school	3.2	6.0	0		
Secondary school	32.2	59.7	0		
> Secondary school (e.g. university)	64.2	33.6	100		
Subjective socioeconomic status (scale 1–10)	6.1 ± 1.8	5.3 ± 1.9	7.0 ± 1.0	< 0.001***^1^	
Work status				< 0.001***^2^	
Paid employment	74.6	56.3	96.1		
Temporarily interrupted paid employment	6.2	8.2	3.9		
Unemployed	19.2	35.5	0		
Household income				< 0.001***^2^	
500–999	1.4	2.6	0^a^		
1.000-1.499	8.9	16.4	0^a^		
1.500-1.999	8.5	12.3	3.9		
2.000-2.499	12.5	18.3	5.7		
2.500-3.999	24.1	27.2	20.5		
4.000-4.999	23.1	11.6	36.7		
> 5.000	21.5	11.6	33.2		
Subjective financial difficulties				< 0.001***^2^	
Very to rather difficult	20.7	38.4	0		
Rather to very easy	79.3	61.6	100		
**Eating behaviour** (Mean +/- SD per day)					
Fruit-and vegetable consumption frequency	2.15 ± 1.15	1.93 ± 1.05	2.40 ± 1.22	< 0.001***^3^	
Fast food consumption frequency	0.20 ± 0.21	0.21 ± 0.24	0.19 ± 0.18	0.39^3^	
Snack consumption frequency	0.72 ± 0.65	0.67 ± 0.61	0.78 ± 0.70	0.21^3^	
Sugar-sweetened beverages consumption frequency	0.42 ± 0.70	0.50 ± 0.77	0.32 ± 0.58	< 0.05*^3^	

FV: fruit-and vegetables, FF: fast-food, HSES: higher socioeconomic status, LSES: lower socioeconomic status, mRFEI: modified retail food environment index, SD: standard deviation

^1^ Assessed using the T.test

^2^ Assessed using the Chi square test

^3^ Assessed using the Mann-Whitney U test

### Differences in food environment along SES

Table 2 shows the characteristics of the respondents’ food environment. Most of the objective and perceived domains of food environment differed between the two groups. Among the objective domains, all indicators related to proximity (i.e., proximity to healthy food outlets, unhealthy food outlets, and fast food outlets) differed significantly between the two groups. This was different for the density metrics. The density of healthy outlets in both the 500m and 1000m buffers did not vary significantly between the groups. The density of unhealthy and fast food outlets varied significantly between LSES and HSES respondents, with a higher density of both types of outlets in the food environment of LSES respondents. Regarding the perceived indicators, all indicators, except the neighborhood availability of fast food, varied significantly between the groups. Respondents from the HSES group in general perceived their food environment as better than respondents from the LSES group.

**Table 2:** Indicators of the objective and perceived food environment of the overall study sample and by SES. Respondents in the CIVISANO-survey, Flanders, 2021

	Total sample (*n* = 498) Mean or %	LSES (*n* = 268)	HSES (*n* = 229)	Test statistic (i.e. T-test/Chi-square/Mann-Whitney U)
**Perceived food environment**				
FV availability				< 0.005**
Disagree/strongly disagree	5.2	8.6	1.3	
Neutral	5.2	7.8	2.2	
Agree/Strongly agree	89.6	83.6	96.5	
FF availability				0.30
Disagree/strongly disagree	5.8	6.8	4.8	
Neutral	18.3	20.1	16.2	
Agree/Strongly agree	75.6	73.1	79.0	
FV price				< 0.005**
Disagree/strongly disagree	37.2	43.3	30.1	
Neutral	36.0	34.0	38.5	
Agree/Strongly agree	26.8	22.7	31.4	
FF price				< 0.005**
Disagree/strongly disagree	26.4	31.3	20.5	
Neutral	38.0	38.4	37.6	
Agree/Strongly agree	35.6	30.3	41.9	
FV quality				< 0.005**
Disagree/strongly disagree	7.6	10.5	4.3	
Neutral	17.1	22.0	11.4	
Agree/Strongly agree	75.3	67.5	84.3	
**Objective food environment**				
Proximity to healthy outlets	1541.7 ± 1080.3	1481.4 ± 1090.9	1612.0 ± 1065.8	< 0.005**
Proximity to unhealthy outlets	561.7 ± 477.0	531.4 ± 476.4	597.2 ± 476.3	< 0.005**
Proximity to FF outlets	760.9 ± 591.5	685.3 ± 549.1	849.5 ± 627.3	< 0.005**
Density of healthy outlets 500m	0.3 ± 0.5	0.3 ± 0.6	0.2 ± 0.5	0.07
Density of unhealthy outlets 500m	2.0 ± 2.5	2.1 ± 2.5	1.9 ± 2.5	0.002**
Density of FF outlets 500m	0.8 ± 1.1	1.0 ± 1.2	0.5 ± 1.0	0.02*
mRFEI 500m	0.04 ± 0.1	0.05 ± 0.09	0.04 ± 0.1	0.07
Density of healthy outlets 1000m	0.2 ± 0.5	0.2 ± 0.5	0.2 ± 0.5	0.08
Density of unhealthy outlets 1000m	2.7 ± 2.8	2.7 ± 2.7	2.8 ± 2.8	< 0.005**
Density of FF outlets 1000m	1.1 ± 1.2	1.1 ± 1.2	1.1 ± 1.2	0.004*
mRFEI 1000m	0.04 ± 0.1	0.04 ± 0.1	0.04 ± 0.1	0.08

FV: fruit-and vegetables, FF: fast-food, HSES: higher socioeconomic status, LSES: lower socioeconomic status, mRFEI: modified retail food environment index

### Food environment, eating behavior and SES

**Fig. 1:**
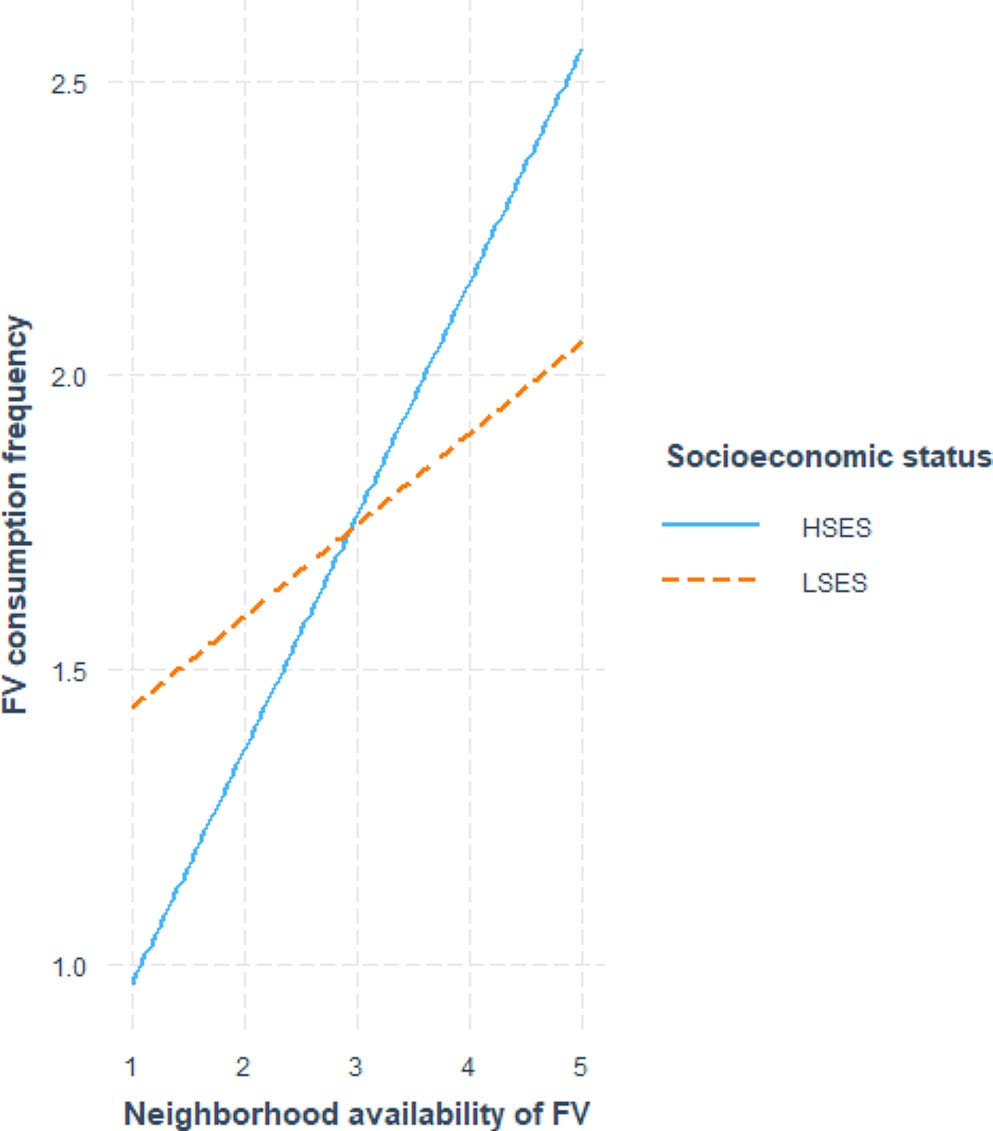
Interaction effect of SES on the perceived availability of FV in the neighborhood for FV consumption frequency per day. FV: fruit-and vegetables, HSES: higher socioeconomic status, LSES: lower socioeconomic status, SES: socioeconomic status

**Fig. 2:**
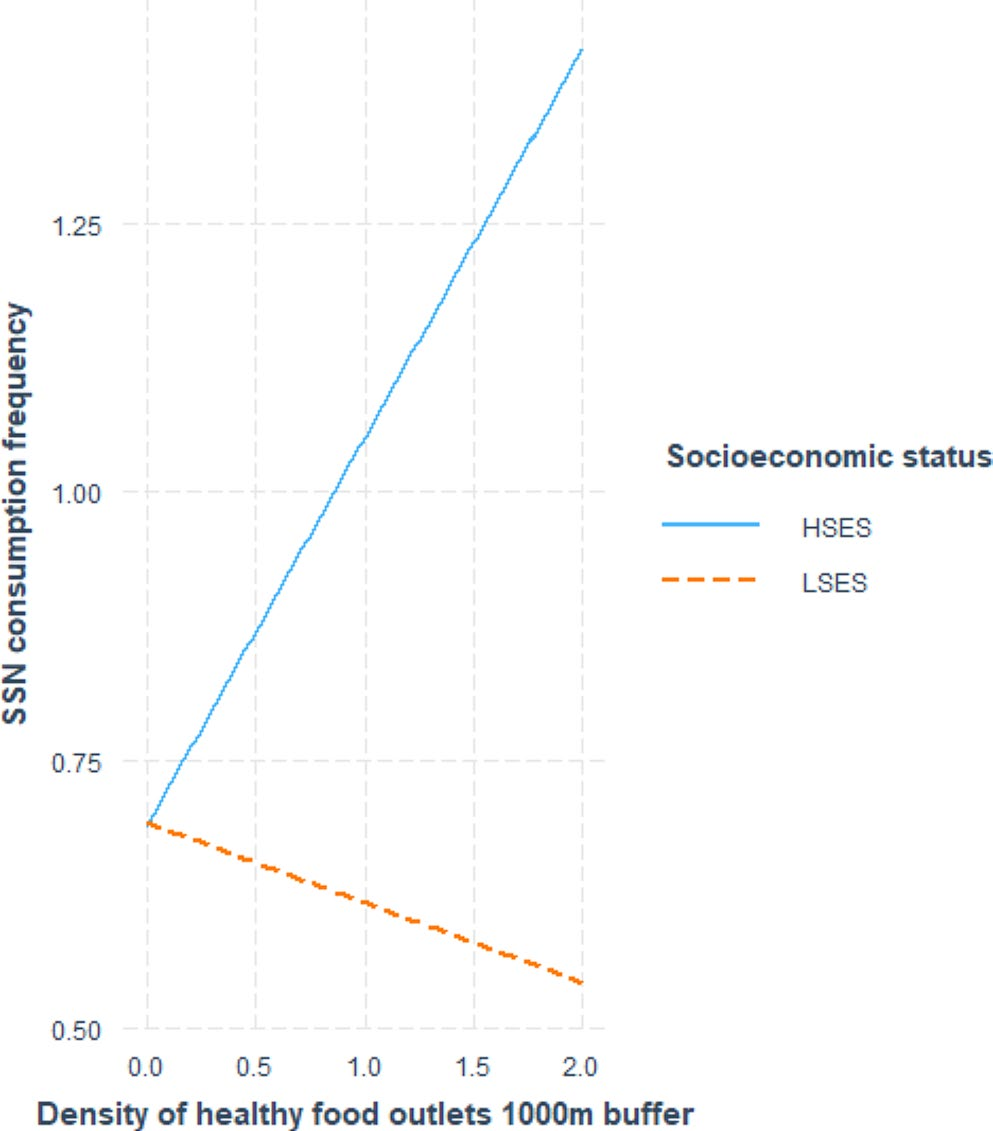
Interaction effect of SES on the density of healthy food outlets in the 1000m buffer for SSN consumption frequency per day. HSES: higher socioeconomic status, LSES: lower socioeconomic status, SES: socioeconomic status, SSN: snacks

**Fig. 3:**
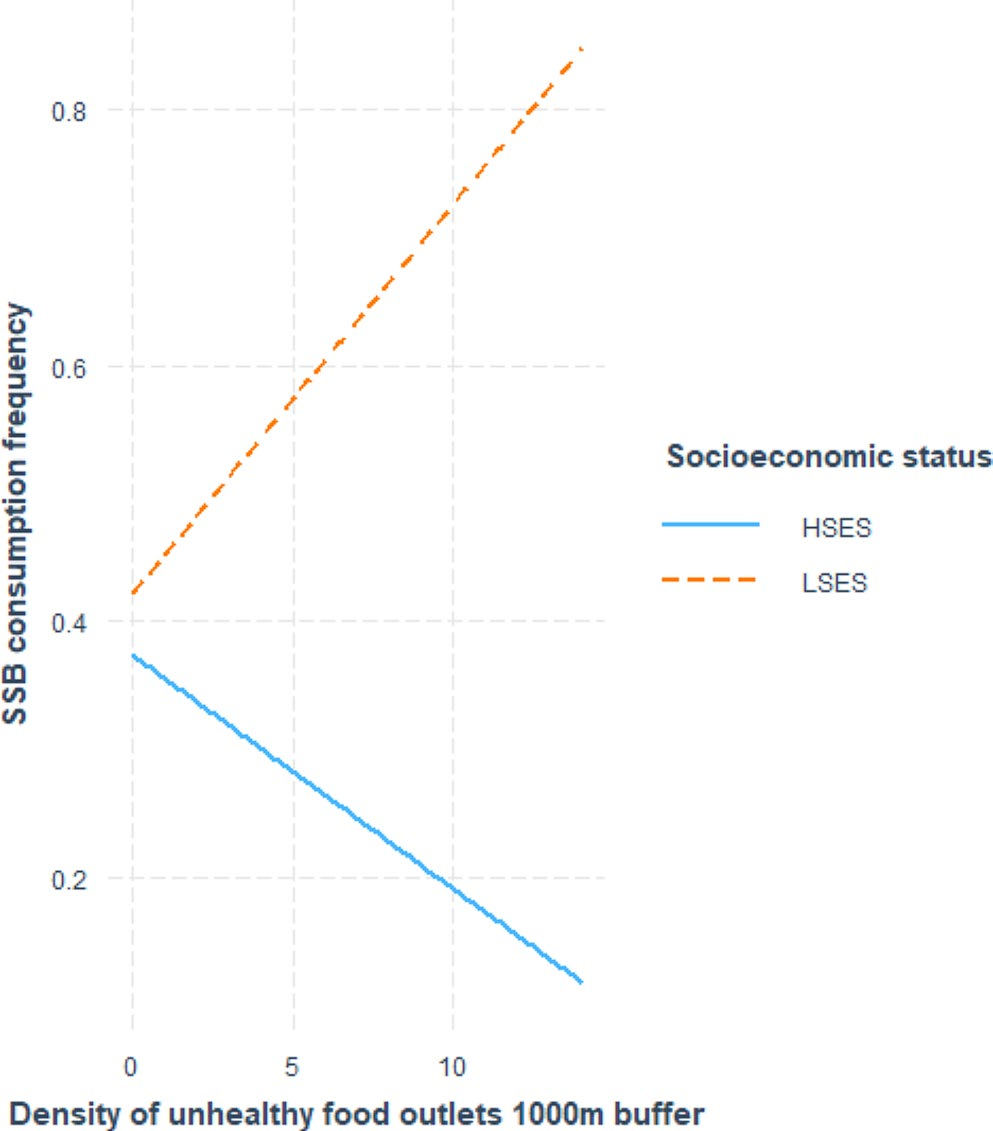
Interaction effect of SES on the density of unhealthy food outlets in the 1000m buffer for SSBs consumption frequency. HSES: higher socioeconomic status, LSES: lower socioeconomic status, SES: socioeconomic status, SSB: sugar-sweetened beverages

Table 3; Figs. 1, 2 and 3 show the associations between indicators of food environment and eating behavior. Overall, two statistically significant main effect was observed between the indicators of food environment and eating behavior. A positive association (β0.26, CI0.15;0.39) was observed between the consumption frequency of fruit and vegetables and the perceived availability of fruit and vegetables in the neighborhood, and a negative association (β-0.03, CI-0.06;-0.003) was observed between fast-food consumption frequency and the density of fast-food outlets in the 500m buffer. The majority of the observations, were those for which SES moderated the associations between objective and perceived food environment and eating behavior, as shown in Table 4. For the association between the perceived availability of FV in the neighborhood and the consumption frequency of FV, statistically significant moderation according to SES was observed (β0.26,CI0.13;0.39). Stratified analysis, visualized in Fig. 1, showed a positive association between availability of FV in the neighborhood and the consumption of FV for the LSES (β0.15, CI0.03;0.27) and HSES-group (β0.37, CI 0.12;0.63). Additionally, several other statistically significant interactions between SES and food environment indicators were observed. SES moderated the association between the density of healthy food outlets in the 1000m buffer and the consumption frequency of snack foods (β-0.60, CI-0.94; -0.23). Stratified analysis, visualized in Fig. 2, did not reveal a statistically significant association in the LSES-group. However, a significant positive association (β0.36,CI 0.18;0.55) was observed in the HSES group; therefore, a higher density of healthy food outlets was associated with a higher consumption frequency of snack foods. A statistically significant interaction was observed between SES and the density of unhealthy food outlets in the 1000m buffer, in relation to the consumption frequency of sugar-sweetened beverages. Through stratified analysis, visualized in Fig. 3, it was established that the association between the density of unhealthy food outlets in the 1000m buffer and the consumption frequency of sugar-sweetened beverages was not significant in the LSES-group. A statistically significant negative association (β-0.02,CI -0.05;-0.0002) was observed in the HSES group. This indicates that in the HSES group, a higher density of unhealthy food outlets was associated with a lower consumption frequency of sugar-sweetened beverages.

**Table 3:** Linear regression models of objective and perceived food environment on eating behavior outcomes (i.e., fruit and vegetables, fast food, snacks, and sugar-sweetened beverage consumption frequency). Respondents in the CIVISANO-survey, Flanders, 2021

	FV β (95% CI)	FF β (95% CI)	SN β (95% CI)	SSB β (95% CI)
**Perceived food environment**			NA	NA
Age	0.005 (-0.004; 0.11)	-0.003 (-0.005; -0.001)***		
Gender identity				
Male	Reference	Reference		
Female	0.42 (0.21; 0.63)**	-0.08 (-0.12; -0.04)***		
Other	0.70 (-0.87; 2.27)	0.04 (-0.25; 0.33)		
FV availability	0.26 (0.15; 0.38)***^a^	NA		
FF availability	NA	0.01 (-0.01; 0.03)		
FV price	0.03 (-0.08; 0.15)	NA		
FF price	NA	0.01 (-0.01; 0.03)		
FV quality	-0.03 (-0.16; 0.11)	NA		
**Objective food environment**				
Age	0.006 (-0.003; 0.02)	-0.003 (-0.01; -0.001)***	-0.005 (-0.01; 0.0001)	-0.01 (-0.02; -0.01)***
Gender identity				
Male	Reference	Reference	Reference	Reference
Female	0.43 (0.21; 0.64)***	-0.1 (-0.12; -0.04)***	0.02 (-0.10; 0.15)	-0.24 (0.37; -0.11)***
Other	0.67 (-0.95; 2.28)	0.02 (-0.27; 0.31)	-0.12 (-1.03; 0.80)	-0.18 (-1.14; 0.78)
Proximity to healthy outlets	-0.0001 (-0.0002; 0.00004)	0.000003 (-0.00002; 0.00003)	0.00002 (-0.0001; 0.0001)	0.000004 (-0.0001; 0.0001)
Proximity to unhealthy outlets	0.00009 (-0.0002; 0.0004)	NA	-0.0001 (-0.0003; 0.00002)	-0.0001 (-0.0003; 0.0001)
Proximity to FF outlets	NA	-0.00001 (-0.0001; 0.00003)	NA	NA
Density of healthy outlets 500m	-0.16 (-0.50; 0.17)	0.04 (-0.03; 0.11)	-0.03 (-0.22; 0.17)	0.03 (-0.17; 0.23)
Density of unhealthy outlets 500m	-0.02 (-0.07; 0.03)	NA	-0.01 (-0.03; 0.02)	-0.05 (-0.23; 0.14)
Density of FF outlets 500m	NA	-0.03 (-0.06; 0.003)	NA	NA
mRFEI 500m	-0.63 (-1.66; 1.10)	-0.15 (-0.41; 0.12)	-0.10 (-0.92; 0.72)	-0.17 (-1.03; 0.69)
Density of healthy outlets 1000m	-0.12 (-0.42; 0.19)	-0.03 (-0.09; 0.03)	0.15^a^(-0.03;0.32)	-0.001 (-0.23; 0.14)
Density of unhealthy outlets 1000m	0.02 (-0.02; 0.06)	NA	0.003 (-0.02; 0.02)	-0.001 (-0.02;0.02)
Density of FF outlets 1000m	NA	0.01 (-0.01; 0.03)	NA	NA
mRFEI 1000m	-0.27 (-1.65; 1.10)	0.03 (-0.22; 0.28)	-0.28 (-1.06; 0.50)	-0.15 (-0.97;0.67)

*p < 0.05,***p < 0.001, ^a^= Indicates significant moderation by SES (*p* < 0.05), NA: not applicable for the eating behaviour outcome, FV: fruit-and vegetables, FF: fast-food, HSES: higher socioeconomic status, LSES: lower socioeconomic status, mRFEI: modified retail food environment index

**Table 4:** Linear regression models with statistically significant interactions between the objective or perceived food environment and eating behavior (i.e. fruit-and vegetables (FV), fast food (FF), snack (SSN) and sugar-sweetened beverages (SSB) consumption frequency) for the whole sample and stratified according to SES. Respondents in the CIVISANO-survey, Flanders, 2021

Indicators of the food environment*SES	Eating behaviour	Total sample β (95% CI)	LSES β (95% CI)	HSES β (95% CI)
FV availability	FV	0.23 (0.03;0.49)*	0.15 (0.03;0.27)*	0.37 (0.12–0.63)**
Density of healthy food outlets 1000m buffer	SSN	-0.60(-0.94; -0.23)**	-0.08 (-0.22;0.07)	0.36 (0.18;0.55)***
Density of unhealthy outlets 1000m buffer	SSB	0.06 (0.02; 0.11)^**^	0.03 (-0.006;0.06)	-0.02(-0.05;-0.0002)*

FV: fruit-and vegetables, HSES: higher socioeconomic status, LSES: lower socioeconomic status, SES: socioeconomic status, SSN: snacks, SSB: sugar-sweetened beverages

## Discussion

This study examined whether the food environment varied by socioeconomic status and whether SES moderated the association between the food environment and eating behavior. First, regarding the variation in food environment by SES, it was found that the food environment differed between HSES and LSES respondents in peri-urban areas. This is not surprising, as differences in the food environment in neighborhoods with different SES profiles have long been described in the literature [31, 32]. However, in this study, both SES and food environment were measured at the individual level rather than at the neighborhood level. When these differences in food environment between SES groups were examined, it was found that for the perceived food environment, respondents from the HSES group perceived greater access to FV in terms of availability, price, and quality than those from the LSES group. This is in line with copious previous research, which has reported differences in perceptions of the food environment between SES groups [60, 61]. This study did not assess what determines perceptions of the food environment and why respondents with LSES had a more negative perception of their food environment than respondents with HSES. Although, as previously states multiple studies have reported these differences in perceptions regarding the food environment, limited work is available on what is behind these differences. Therefore, this topic warrants further research and it is recommended that future research assess why perceptions of the food environment differ between groups with diverging SES profiles. It may be that these differences are influenced by the ability of people with HSES to more easily choose where to live due to less financial constraints, compared to people with LSES who may be more bound to neighborhoods that are affordable to them.

For the objective food environment, proximity indicators differed between SES groups. Respondents with LSES had greater proximity to healthy, unhealthy, and fast food outlets. The associations between objective and perceived food environment domains and eating behavior were also assessed. Here, only one main effect between food environment and eating behavior was observed, a negative association between FF consumption frequency and the density of FF outlets in the 500m buffer. This is partly in line with earlier studies, which have reported statistically insignificant associations between the domains of food environment and eating behavior [33, 34]. Suggesting that the overall link between the food environment, especially the objective domain and eating behavior, might be more complex than initially thought and that the concept of objective indicators of the food environment representing the type and location of food outlets on people’s eating behavior is not enough to provide a holistic view of the food environment, to which an individual is exposed. This might be because the food environment is a vastly complex system and that focusing on a single setting, such as the neighborhood setting of this study, narrows the food environment to an ´artificial environment´ and deviates from the complexity of real-life food acquisition and eating behavior, resulting in statistically insignificant observations [35].

Associations were mainly observed where socioeconomic status moderated the impact of the food environment on eating behavior, underscoring differences across socioeconomic profiles. Not considering this difference might result in a conclusion that has reduced applicability to people in specific socioeconomic positions. Resulting food policies would not be applicable to all population groups and could cause adverse effects for those most in need of healthy food environments [36]. Moderation was not observed for each association between the food environment and eating behaviour, it was only observed for three associations, i.e. for the perceived availability of fruit and vegetables in the neighborhood and fruit and vegetables consumption frequency, for the density of healthy food outlets in the 1000m buffer and snack consumption and lastly for the density of unhealthy food outlets in the 1000m buffer and the consumption frequency of sugar-sweetened-beverages. These associations were noted for fruit-and vegetable consumption frequency, sugar-sweetened-beverages consumption frequency, and snack consumption frequency, but not for fast food consumption frequency. Challenges in defining fast-food purchasing locations may contribute to mixed results across the literature, as this can take place in various locations such as convenience stores or fast-food restaurants, and the purchase location might actually be setting specific; in an urban setting, fast-food consumption might more often occur in a fast-food restaurant, while in a peri-urban or rural setting, this might be in another type of food outlet because fast-food restaurants might be less prevalent in these settings. Our study lacked chain fast-food restaurants, potentially influencing findings given their association with fast-food consumption [19]. Regarding snack consumption, SES moderated the effect of density within the 1000m buffer. When the groups were examined separately, the association was only significant in the HSES group. This indicates that a higher density of healthy food outlets within the 1000m buffer is associated with a higher consumption of snacks. Both SES groups resided in similar locations; that is, for both groups, the majority of respondents lived around the center of the municipality, and a minority was more dispersed among its fringes. Therefore, the densities of the healthy and unhealthy outlets were similar in both the groups. A plausible explanation may be that respondents with HSES were more often employed than respondents with LSES, and due to this they might take less time to eat, so snacking might replace sitting down to eat. The perception of the availability or accessibility of snacks by SES was an indicator that was not examined in this study, and could provide more insights into this association. Lastly, an interaction between the consumption frequency of SSBs, SES and the density of unhealthy food outlets in the 1000m buffer was observed. After stratified analysis, it was observed that the association was significant only for the HSES. Thus, for the HSES group, a higher density of unhealthy food outlets were associated with a lower consumption frequency of SSBs. This contrasts with prior studies, possibly due to differences in supermarket inclusion and also since SSBs are reportedly most often bought at convenience stores; this type of food outlet is scarce in peri-urban settings [40].

The first strength of this study was the relatively large sample size of people with lower SES. Another strength is that the study was conducted in a peri-urban setting rather than an urban setting, which is more dominant in the European literature related to the food environment. Furthermore, we were able to localize respondents and thus analyze the objective food environment at the individual level rather than using data aggregated at the statistical sector level (i.e., the smallest geographical unit in Belgium), which in peri-urban and rural settings often includes larger areas that may encompass multiple neighborhoods. Therefore, we gained more data precision by analyzing the objective food environment of each respondent. However, this does not represent the “true” food environment of the respondents, since we did not measure the individual food environment of each respondent. An additional strength is that we were able to include multiple indicators of socioeconomic status rather than focusing only on educational attainment, which is often used as the sole proxy for SES.

However, this study has some limitations. First, because of the cross-sectional design of the study, it was not possible to draw firm conclusions about the causal relationship between the objective and perceived food environment, eating behavior, and the underlying moderation by SES. Second, the objective and perceived food environments were solely measured in the neighborhood of residence of the respondents and did not reflect daily real exposure to the food environment. Third, the classification of LSES might lead to a risk of under-adjustment since there is a variability in how many respondents meet one or multiple criteria of LSES. When LSES is based on two criteria for example education and subjective socioeconomic status, but not on additional ones, it could be that the additional criteria, which do not impact LSES, might impact the explanatory variables and eating behaviour outcomes under study. Additionally, data on eating behavior were self-reported by the respondents, and it is well known that self-reported measures are not able to perfectly reflect food consumption, especially over a longer period such as three months and can lead to a social approval bias [41, 42]. Furthermore, data were collected only on the consumption frequency of the selected food groups, and the intake of broader foods and drinks was not considered.

## Conclusion

This study investigated variations in the food environments of people with lower and higher SES, and assessed whether associations between the objective and perceived domains of the food environment and eating behavior were moderated by SES. Both domains of the food environment diverged between SES groups. In general, people with HSES had a more positive perception regarding the availability, quality, and price of FV in their neighborhood than respondents with LSES. In addition, the proximity to healthy, unhealthy, and fast-food outlets was higher for respondents with an LSES than for those with an HSES. For density metrics, no variations between the groups were observed. One main effect between the domains of food environment and eating behavior was observed between FF consumption frequency and the density of FF outlets in the 500m buffer. Multiple instances of moderation according to SES were observed for several eating behaviors, except for the frequency of fast food consumption. Our results underscore the importance of SES as a critical factor in understanding the link between the food environment and eating behavior. This highlights the need for policymakers to consider these findings (i.e., people with lower SES generally have a more negative perception regarding the availability, quality, and pricing of fruits and vegetables in their neighborhoods) when implementing interventions regarding the food environment, especially when interventions on the objective food environment are intended in the neighborhood without considering residents’ perceptions. Furthermore, it is also recommended that future studies on food environment and eating behavior should consider SES, since not taking it into consideration is likely to have an impact on study findings. Stratified analysis in this study showed non-significant associations for the LSES-group, suggesting that the link between the food environment and eating behavior is more pronounced in the HSES-group or that objective indicators might be a proxy for other environmental characteristics that we did not study, such as the in-store availability/affordability of food items. In this study, only a few perceived variables were tested and these indicators were solely related to healthy eating. It is recommended that subsequent studies expand on this, include more perceived indicators, and extend their focus to unhealthy eating behaviors.

### Electronic supplementary material

Below is the link to the electronic supplementary material.

Supplementary Material 1

## Data Availability

The authors do not have permission to share the data.

## Abbreviations

CI: confidence interval
FV: fruit-and vegetables
FF: fast-food
HSES: people with higher socioeconomic status
LSES: people with lower socioeconomic status
mRFEI: modified retail food environment index
SES: socioeconomic status
SSBs: sugar-sweetened beverages
SN: snack foods
