# Supplementary material for: Associations between the objective and perceived food environment and eating behavior in relation to socioeconomic status among adults in peri-urban settings: results from the CIVISANO study in Flanders, Belgium
Source: Int J Health Geogr. 2024 May 9;23:10. doi: 10.1186/s12942-024-00369-4 (PMC11080110; doi:10.1186/s12942-024-00369-4)
Supplement: Supplementary file 1 — Supplementary Material 1 [file 12942_2024_369_MOESM1_ESM.docx]

# Appendix A.

# *Additional file 1:*

Table S1. Representation of indicators of the objective and perceived food environment domains which were included in the multivariable models for each eating behaviour outcome

| **Dependent variables** | **FV** | **FF** | **SN** | **SSB** |
| --- | --- | --- | --- | --- |
| **Explanatory variables** |  |  |  |  |
| **Perceived food environment** |  |  |  |  |
| FV availability | X |  |  |  |
| FF availability |  | X |  |  |
| FV price | X |  |  |  |
| FF price |  | X |  |  |
| FV quality | X |  |  |  |
| **Objective food environment** |  |  |  |  |
| Proximity to healthy outlets | X | X | X | X |
| Proximity to unhealthy outlets | X |  | X | X |
| Proximity to FF outlets |  | X |  |  |
| Density of healthy outlets 500m | X | X | X | X |
| Density of unhealthy outlets 500m | X |  | X | X |
| Density of FF outlets 500m |  | X |  |  |
| mRFEI 500m | X | X | X | X |
| Density of healthy outlets 1000m | X | X | X | X |
| Density of unhealthy outlets 1000m | X |  | X | X |
| Density of FF outlets 1000m |  | X |  |  |
| mRFEI 1000m | X | X | X | X |

FV: fruit-and vegetables, FF: fast-food, mRFEI: modified retail food environment index

# *Additional file 2: Recalculation of consumption frequency values of fruit-and vegetables, fast-food, snacks and sugar-sweetened beverages*

*Table S2: transformation of food consumption frequency values from categorical to numerical in times per day.*

| **Original values (categories)** | | **Calculation steps** | **Converted values (times per day)** |
| --- | --- | --- | --- |
| 1 | Never | 0 | 0 |
| 2 | Less than once per month | < 1/30 times per day → 0 - 0.033 times per day→ average 0.033/2 = 0.017 per day | 0.017 |
| 3 | 1-3 times per month | 1/30 – 3/30 times per day → 0.033 – 0.1 times per day → on average (0.033+0.1)/2 = 0.067 times per day | 0.067 |
| 4 | once per week | 1/7 times per day = 0.14 times per day | 0.14 |
| 5 | 2-4 times per week | 2/7 – 4/7 times per day → 0.29 – 0.57  times per day → on average (0.29 + 0.57)/2 = 0.43 times per day | 0.43 |
| 6 | 5-6 times per week | 5/7 – 6/7 times per day → 0.71 -0.86 times per day → on average (0.71 +  0.86)/2 = 0.79 per day | 0.79 |
| 7 | once per day | 1 time per day | 1 |
| 8 | 2-3 times per day | on average (2+3)/2 = 2.5 times per day | 2.5 |
| 9 | more than 3 times per day | 3 times per day | 3 |
| -1 | missing | NA | NA |

## *Additional file 3: Reclassification of the Locatus food retail outlet categories*

*Table S3: The original food retail categories from the Locatus database and their reclassification for the purposes of this study*

| Reclassification | Locatus classification | Locatus definition |
| --- | --- | --- |
| Fastfood /takeaway/delivery outlets | 59.210.171-Fastfood | Meal provider where service is not at the table, without fixed cutlery and where the usually fried products are ready for consumption within minutes of ordering (excludes sandwich stores) |
|  | 59.210.215-Grillroom/ Shoarma | Sales of grill products, shoarma, kebab and alike. |
|  | 59.210.180-Takeaway/ delivery | Provision of (hot) meals, which are not consumed on site but are collected or delivered |
| Full service restaurants | 59.210.235-Hotel-Restaurant | Hotel combined with ‘a la carte’ restaurant |
|  | 59.210.333-Lunchroom | Meal provider, with table service, particularly including breakfasts, lunches and desserts, opposed to restaurants mostly closed in the evening |
|  | 59.210.430-Café-Restaurant | Provision of both beverages and meals |
|  | 59.210.434-Restaurant | Provision of meals, beverages are provided only in conjunction with the food |
|  | 59.210.392-Pancakes | Restaurants specializing in pancakes |
| Supermarkets | 11.010.519-Supermarket | Stores with a wide and varied range of food products often supplemented by a narrow and shallow range of non-food products. Floor space is greater than 149m² |
| Greengrocers | 11.010.012-Vegetables/ fruit | Almost exclusively sales of potatoes, vegetables and fruit that have not been cultivated by the company itself |
| Shops selling animal products | 11.010.471-Butcher | Sales of meat and meat products |
|  | 11.010.399-Poulterer | Sales of game and poultry |
|  | 11.010.588-Fish | Sales of fish, crustaceans and molluscs |
| Bakeries | 11.010.111-Baker | Sales of bread and pastries, with possible lunchroom but that is not be the main activity |
|  | 11.010.112-Flans | Sales of pastries, with an emphasis on flans |
| Other shops | 11.010.132-Chocolate | Sales of Chocolate |
|  | 11.010.261-Cheese | Sales of Cheese |
|  | 11.010.310-Farm store | Farm sales of own products, primarily food products, supplemented by an assortment of purchased products |
|  | 11.010.378-Nuts | Sales of nuts, dates and dried fruits |
|  | 11.010.423-Reform | Biodynamic, ecological and macrobiotically grown products supplemented with dietary supplements, homeopathic remedies, herbs |
| Convenience stores | 11.010.309-Minisuper | See definition of supermarket, but store is up to 149m² |
|  | 11.010.350-Nightshop | Mini supermarket with (late) evening and night opening |
| Confectionary stores | 11.010.657-Sweets | Sales of sweets and confectionery, also sugars |
|  | 59.210.246-Ice cream parlor | Sales of ice cream |
| Excluded from the study | 11.010.137-Coffee/Tea | Store selling mainly coffee and tea, possibly supplemented by items intended for drinking coffee/tea |
|  | 11.010.424-Food Supplements | Sales of supplements intended to supplement or replace a normal diet. Also vitamins, sports supplements |
|  | 11.010.141-Delicacies | Store that sells specialty, usually more luxurious foods, and often convenience products |
|  | 11.010.477-Liquor | Sales of alcoholic and non-alcoholic beverages |
|  | 59.210.123-Bar | Provision of almost exclusively beverages (not meals) |
|  | 59.210.127-Coffee house | Provision of only non-alcoholic beverages (in the regulation Turkish or Moroccan beverages) |
|  | 59.210.334-Coffee bar | Provision of predominantly (hot, non-alcoholic) beverages prepared by a barista, limited offering of breakfasts, lunches and desserts. Predominantly ordering at the counter. |
|  | 59.210.950-Horeca other | Hospitality industry not further classifiable into one of the other categories |
|  | 11.010.123-Toko | Store selling mainly oriental foods |

## *Additional file 4 – Health scores based on expert opinion*

*Table S4: Health scores of the food outlets in the Locatus database based on the expert opinion of 15 Flemish experts*

| **Locatus Category** | **Explanation** | **Likert Scale Score** | **Healthy/neutral/unhealthy** |
| --- | --- | --- | --- |
| **Primary food retailers** |  |  |  |
| 59.210.171-Fastfood | Meal provider where service is not at the table, without fixed cutlery and where the usually fried products are ready for consumption within minutes of ordering (excludes sandwich stores). | 1 | Unhealthy |
| 59.210.215-Grillroom/Shoarma | Sales of grill products, shoarma, kebab and alike. | 1 | Unhealthy |
| 59.210.180-Delivery | Provision of (hot) meals, which are not consumed on site but are collected or delivered. | 2 | Unhealthy |
| 59.210.235-Hotel-Restaurant | Hotel combined with ‘a la carte’ restaurant. | 3 | Neutral |
| 59.210.333-Lunchroom | Meal provider, with table service, particularly including breakfasts, lunches and desserts, opposed to restaurants mostly closed in the evening. | 3 | Neutral |
| 59.210.430-Café-Restaurant | Provision of both beverages and meals. | 2 | Unhealthy |
| 59.210.434-Restaurant | Provision of meals, beverages are provided only in conjunction with the food. | 3 | Neutral |
| 59.210.392-Pancakes | Restaurants specializing in pancakes. | 2 | Unhealthy |
| 11.010.519-Supermarkt | Stores with a wide and varied range of food products often supplemented by a narrow and shallow range of non-food products. Floor space is greater than 149m². | 3 | Neutral |
| 11.010.309-Minisuper | See definition of supermarket, but store is up to 149m². | 3 | Neutral |
| 11.010.012-Greengrocer | Almost exclusively sales of potatoes, vegetables and fruit that have not been cultivated by the company itself. | 5 | Healthy |
| 11.010.471-Butcher | Sales of meat and meat products. | 2 | Unhealthy |
| 11.010.399-Poulterer | Sales of game and poultry. | 3 | Neutral |
| 11.010.588-Fish | Sales of fish, crustaceans and molluscs. | 4 | Healthy |
| 11.010.261-Cheese | Sales of Cheese. | 3 | Neutral |
| 11.010.111-Bakery | Sales of bread and pastries, with possible lunchroom but that is not be the main activity. | 3 | Neutral |
| 11.010.112-Flans | Sales of pastries, with an emphasis on flans. | 2 | Unhealthy |
| 11.010.310-On Farm Store | Farm sales of own products, primarily food products, supplemented by an assortment of purchased products. | 4 | Healthy |
| 11.010.378-Nuts | Sales of nuts, dates and dried fruits. | 4 | Healthy |
| 11.010.423-Bio Store | Biodynamic, ecological and macrobiotically grown products supplemented with dietary supplements, homeopathic remedies, herbs. | 4 | Healthy |
| 11.010.132-Chocolate | Sales of Chocolate. | 1 | Unhealthy |
| 11.010.657-Candystore | Sales of sweets and confectionery, also sugars. | 1 | Unhealthy |
| 59.210.246-Icecream Parlor | Sales of ice cream. | 1 | Unhealthy |
| 11.010.350-Nightshop | Mini supermarket with (late) evening and night opening. | 2 | Unhealthy |
|  |  |  |  |
| **Secondary food retailers** |  |  | Unhealthy |
| 11.010.522-Tobacco | Sale of tobacco products and smoking accessories, in conjunction with magazines, confectionery and soft drinks. | 1 | Unhealthy |
| 11.020.156-Drugstore | Sells personal care products, self-help medications, health care products, nursing and cleaning supplies. | 2 | Unhealthy |
| 22.030.618-Warehouse | Store with a broad assortment of alimentary and non-alimentary products in which the fashion segment is often predominant. | 2 | Unhealthy |
| 45.205.528-Gas station | Sale of automotive fuels, with or without a store. | 2 | Unhealthy |
| 59.210.150-Disco | Opportunity for evening-night catering with a central dance floor and loud music. | 1 | Unhealthy |
| 59.210.155 Sex/partyclubs | Opportunity for sex, erotic massages, shows, couples clubs etc. | 1 | Unhealthy |
| 59.210.465-Partycenter | Rental of rooms for parties and celebrations, including the provision of food and beverages for these parties. | 2 | Unhealthy |
| 59.230.018-Amusement hall | Is an age-independent, freely accessible place with gambling and gaming machines. | 1 | Unhealthy |
| 59.230.020-Theme park | Terrain on which one or more attractions are assembled. | 2 | Unhealthy |
| 59.230.028-Casino | An adult-only venue where you can play slot machines and various gambling games such as roulette and blackjack at a table in luxurious surroundings. | 1 | Unhealthy |
| 59.230.078-Biljart/Pool | Space primarily designed for billiards/pool and / or snooker (any catering service here is additional and not the main purpose). | 2 | Unhealthy |
| 59.230.080-Indoor playground | Indoor play area for children. | 2 | Unhealthy |
| 59.230.102-Bowling | Bowling center (catering service is complementary here and not the main purpose). | 2 | Unhealthy |
| 59.230.150-Zoo | Grounds where animals can be viewed for a fee. | 2 | Unhealthy |
| 59.230.950-Amusement Other | All other forms of entertainment, not previously mentioned. | 2 | Unhealthy |
| 65.250.033-Video Store | Rental of image and sound carriers with or without computer games. | 1 | Unhealthy |
| 59.220.081-Cinema | Screening of films. | 1 | Unhealthy |
| 59.220.549-Theater | Room for the screening of live shows. | 2 | Unhealthy |
| 59.230.265-Go Cart Track | Place where karts can be rented and driven . | 2 | Unhealthy |
| 59.230.295-Ice Skating Track | Permanently established indoor ice rink where artificial ice can be skated for a fee. | 2 | Unhealthy |
| 59.230.290-Climbing Hall | Indoor facility for the practice of climbing . | 2 | Unhealthy |
| 59.230.310-Laser Game | Center where laser games can be played. | 2 | Unhealthy |
| 59.230.590-Ski Track | Indoor place where skiing can be done. | 2 | Unhealthy |
| 59.230.700-Swimming Pool | Indoor public pool. | 2 | Unhealthy |
| 59.230.570-Sauna | Sauna complex accessible to everyone. | 2 | Unhealthy |

*Additional file 5 – Analysis of the main effects between indicators of the objective and perceived food environment on eating behaviour (i.e. fruit-and vegetable (FV), fast-food (FF), snacks (SN), sugar-sweetened beverages (SSB) consumption frequency) adjusted for the five indicators of SES (i.e. education, subjective SES, employment status, income, subjective financial status)*

*Table S5: The main effect of indicators of the objective and perceived food environment on eating behaviour adjusted for income, subjective SES, employment status, income and subjective financial status*

|  | **FV**  β (95% CI) | **FF**  β (95% CI) | **SN**  β (95% CI) | **SSB**  β (95% CI) |
| --- | --- | --- | --- | --- |
| **Covariates** |  |  |  |  |
| Age | 0.02 (0.01 ;0.025) | -0.003 (-0.01; -0.002) | -0.01 (-0.01; -0.0001) | -0.01 (-0.02; -0.01) |
| Gender identity |  |  |  |  |
| Male | Reference | Reference | Reference | Reference |
| Female | 0.42 (0.21; 0.64) | -0.1 (-0.11; -0.04) | 0.05 (-0.08; 0.18) | -0.22 (-0.35; -0.09) |
| Other | 0.97 (-0.60; 2.54) | -0.01 (-0.30; 0.29) | -0.03 (-0.96; 0.90) | -0.12 (-1.07; 0.83) |
| **SES variables** |  |  |  |  |
| Education |  |  |  |  |
| None | -0.12 (-1.71; 1.47) | 0.05 (-0.25; 0.35) | -0.56 (-1.49; 0.38) | -0.09 (-1.04; 0.86) |
| Primary education | -0.19 (-0.78; 0.40) | -0.01 (-0.12; 0.10) | -0.07 (-0.42; 0.27) | 0.42 (0.07; 0.77) |
| Secondary education | -0.43***(-0.68; -0.19) | 0.06**( 0.01; 0.11) | 0.06 (-0.08; 0.20) | 0.24 (0.09; 0.39) |
| Higher education | Reference | Reference | Reference | Reference |
| Subjective SES |  |  |  |  |
| <5 | -0.05 (-0.36; 0.26) | 0.03 (-0.02; 0.09) | -0.06 (-0.24; 0.12) | -0.12 (-0.31; 0.06) |
| >5 | Reference | Reference | Reference | Reference |
| Employment status |  |  |  |  |
| Paid employment | Reference | Reference | Reference | Reference |
| Unemployment | -0.05 (-0.48; 0.38) | -0.07 (-0.15; 0.01) | 0.08 (-0.17; 0.34) | 0.16 (-0.09; 0.42) |
| Temporary unemployed | -0.09 (-0.42; 0.23) | -0.027 (-0.1; 0.03) | 0.15 (-0.05; 0.34) | 0.11 (-0.08; 0.31) |
| Income |  |  |  |  |
| <1.499 | -0.42 (-0.89; 0.05) | 0.05 (-0.04; 0.13) | -0.07 (-0.35; 0.21) | 0.30 (0.01; 0.58) |
| 1500-2499 | -0.33*( -0.65;-0.01) | 0.02 (-0.04; 0.08) | -0.12 (-0.30; 0.01) | 0.02 (-0.17; 0.21) |
| 2.500-3.999 | -0.04 (-0.30; 0.22) | 0.027 (-0.02; 0.08) | -0.14 (-0.35; 0.01) | -0.01 (-0.17; 0.15) |
| >4.000 | Reference | Reference | Reference | Reference |
| Subjective financial status |  |  |  |  |
| Very difficult to rather difficult | -0.17 (-0.49; 0.12) | 0.01 (-0.05; 0.07) | -0.17 (-0.35; 0.01) | 0.05 (-0.13; 0.24) |
| Very easy to rather easy | Reference | Reference | Reference | Reference |
| **Perceived food environment** |  |  |  |  |
| FV availability | 0.03  (-0.19; 0.25) |  |  |  |
| FF availability |  | 0.01 (-0.02; 0.05) |  |  |
| FV price | 0.04  (-0.09;0.18) |  |  |  |
| FF price |  | 0.02 (-0.01; 0.04) |  |  |
| FV quality | -0.02  (-0.20 ;0.17) |  |  |  |
| **Objective food environment** |  |  |  |  |
| Proximity to healthy outlets | -0.0001 (-0.0003; 0.00001) | 0.00001 (-0.00002; 0.00004) | 0.00002 (-0.0001; 0.0001) | 0.00002 (-0.00006; 0.0001) |
| Proximity to unhealthy outlets | 0.0001 (-0.0002; ;0.0004) |  | -0.0002 (-0.0003; 0.000003) | -0.0001 (-0.0003; 0.00003) |
| Proximity to FF outlets |  | -0.00001 (-0.00006086254 0.00003) |  |  |
| Density of healthy outlets 500m | -0.05 (-0.39 ;0.28) | 0.05 (-0.03; 0.13) | 0.03 (-0.16; 0.23) | 0.005 (-0.19; 0.21) |
| Density of unhealthy outlets 500m | -0.02 (-0.07 ;0.03) |  | -0.01 (-0.0002; 0.35) | -0.02 (-0.05; 0.01) |
| Density of FF outlets 500m |  | -0.03* (-0.06;-0.003) |  |  |
| mRFEI 500m | -0.79 (-0.22 ;0.63) | -0.16 (-0.42; 0.10) | -0.20 (-1.03; 0.63) | -0.24 (-1.1; 0.61) |
| Density of healthy outlets 1000m | -0.14 (-0.44 ;0.16) | -0.02 (-0.08: 0.04) | 0.18 (-0.0002; 0.35) | -0.03 (-0.21; 0.15) |
| Density of unhealthy outlets 1000m | 0.01 (-0.03 ;0.05) |  | 0.002 (-0.02; 0.02) | -0.001 (-0.02; 0.02) |
| Density of FF outlets 1000m | -0.36 (-1.70 ;0.97) | 0.01 (-0.01; 0.03) |  |  |
| mRFEI 1000m | -0.12 (-1.70;0.97) | 0.03 (-0.22; 0.28) | -0.40 (-1.19; 0.39) | -0.20 (-1.05; 0.86) |
